# Measuring the impacts of adaptation strategies to drought stress: The case of drought tolerant maize varieties

**DOI:** 10.1016/j.jenvman.2017.06.058

**Published:** 2017-12-01

**Authors:** Tesfamicheal Wossen, Tahirou Abdoulaye, Arega Alene, Shiferaw Feleke, Abebe Menkir, Victor Manyong

**Affiliations:** a International Institute of Tropical Agriculture (IITA), Abuja, Nigeria; b IITA, Ibadan, Nigeria; c IITA, Lilongwe, Malawi; d IITA, Dar es Salaam, Tanzania

**Keywords:** Downside risk, Drought tolerant maize varieties, Endogenous switching regression, Nigeria, Productivity, Welfare

## Abstract

This study measured the impacts of drought tolerant maize varieties (DTMVs) on productivity, welfare, and risk exposure using household and plot-level data from rural Nigeria. The study employed an endogenous switching regression approach to control for both observed and unobserved sources of heterogeneity between adopters and non-adopters. Our results showed that adoption of DTMVs increased maize yields by 13.3% and reduced the level of variance by 53% and downside risk exposure by 81% among adopters. This suggests that adoption had a “win-win” outcome by increasing maize yields and reducing exposure to drought risk. The gains in productivity and risk reduction due to adoption led to a reduction of 12.9% in the incidence of poverty and of 83.8% in the probability of food scarcity among adopters. The paper concluded that adoption of DTMVs was not just a simple coping strategy against drought but also a productivity enhancing and welfare improving strategy. The results point to the need for policies and programs aimed at enhancing adoption as an adaptation strategy to drought stress in Nigeria and beyond.

## Highlights

• Impact of drought tolerant maize varieties evaluated.
• Drought tolerant maize varieties increased productivity and reduced downside risk.
• Drought tolerant maize varieties reduced the incidence of poverty.
• Mainstreaming drought tolerant maize varieties into national climate change adaptation plans will be important.

## 1 Introduction

Agriculture in Africa is highly vulnerable to climate change and variability (Schlenker and Lobell, 2010, Haile et al., 2017). The occurrence of climate change-induced rainfall shock in general and drought shock in particular affects food security in many developing countries (Wossen et al., 2016). As a result of climate change, droughts have become more severe, longer, and more frequent (Hyman et al., 2008). The economic costs can, therefore, be enormous as drought has the potential to cause a severe food crisis, hunger and malnutrition, as well as sustained long-term poverty traps due to the limited adaptive capacity of smallholders (Collier et al., 2008, Bryan et al., 2013). Of particular interest, at least in the context of Africa, is the adverse effect of drought on the production of maize, Africa's most important food crop. Maize is grown on nearly 30 million ha of land, supporting over 300 million people on the continent (Tambo and Abdoulaye, 2012, La Rovere et al., 2014, Fisher et al., 2015). However, maize is also a crop that is highly susceptible to drought. According to Fisher et al. (2015), around 40% of Africa's maize-growing areas face occasional drought stress, resulting in yield losses of 10–25%. Moreover, Schlenker and Lobell (2010) pointed out that production of maize would decline by 22% in sub-Saharan Africa (SSA) by 2050 due to climate change. Reducing the vulnerability of maize producers to drought shocks is, therefore, an important entry point to improve productivity and hence reduce the prevalence of food insecurity and poverty.

Efforts have been made to develop adaptation strategies against drought stress. Notable among these was the DTMA project - Drought Tolerant Maize for Africa - which was initiated with the aim of developing and deploying drought-tolerant maize varieties (DTMVs). As the project targeted production zones where the rainfall patterns and climatic conditions varied considerably within and among seasons, the varieties that were developed were selected for high yield potential under both drought stress and favourable growing conditions. Over 200 distinct DTMVs were released in 13 countries across SSA with the support of the DTMA project in the last nine years (Fisher et al., 2015). These varieties were endowed not only with tolerance to drought but also with high levels of lysine and tryptophan, better nitrogen use-efficiency and resistance to the major foliar diseases (Fisher et al., 2015). Adoption will therefore be crucial as it might reduce the variance and downside risk (probability of crop failure) associated with maize production.

A drought shock, besides exacerbating current levels of food insecurity, may lead to sustained long-term asset poverty traps as poor farmers may sell their key assets, such as land and livestock, as a coping measure. In addition, drought-induced crop failures can adversely affect labour supply to agricultural production, education, and health outcomes. The lack of formal insurance and social safety nets in many African countries implies that the risk of drought can be consequential and that variance and downside risk-reducing technologies can provide substantial gains for poor and food insecure farmers (Kostandini et al., 2013). As such, DTMVs can serve as a risk reducing technology option in the absence of formal insurance and safety net mechanisms (La Rovere et al., 2014, Fisher et al., 2015). In doing so, they will enhance food security while acting as an insurance against crop failure. However, empirical evidence on this insurance function is non-existent.

As production risk is the inherent feature of African agriculture, investigating the risk reducing effects of DTMVs is one of the objectives of this paper. We considered risk exposure in addition to productivity as both the variability and skewness of maize yield affect adoption decisions. In this context, besides their effect on productivity, DTMVs can generate benefits by reducing farmers' exposure to risk in general and downside risk in particular.^1^ Since any firm economic understanding of the potential roles of DTMVs under climate change and variability requires an understanding of the dynamics and cross-sectional patterns of adoption, the paper also examined the main determinants of adoption as well as the potential benefits associated with it. The main contributions of this paper are twofold: (1) to investigate how adoption affected productivity as well as exposure to drought risk by explicitly estimating its effect on the variance and skewness of maize yields; and (2) to assess the effects of adoption on household food security and poverty. The rest of the paper is organized as follows. Section 2 presents the conceptual framework. The data and descriptive statistics as well as the empirical estimation strategy are presented in Section 3. The results are presented in Section 4. Section 5 concludes with implications for policy.

## 2 Conceptual framework

Following Koundouri et al. (2006), we investigated the underlying effects of production risk and the risk-mitigating role of DTMVs within the expected utility framework. In particular, we used the moment-based (Antle, 1983) approach which enables the flexible estimation of a stochastic production function under uncertainty. Consider a typical maize producing farmer with a production function *y* = *g* (**x**, ***s***, **w, e**), where ***y*** is maize output, **x** is a vector of inputs other than DTMVs, **s** represents improved seeds (in this case, DTMVs), **w** is weather variables, ***e*** is a vector of village fixed effects, and *g* (**x**, ***s***, **w, e**) represents the corresponding production technology, given **x, s, e,** and **w**. We assume that the production function is strictly concave and twice differentiable with the usual conditions $\;g^{\prime }\left(x,s,w,e\right)>0\;and\;g^{\prime \prime }\left(x,s,w,e\right)<0$. Furthermore, suppose that a typical farmer acquires input ***x*** with a unit cost of ***r*** and DTMVs with a unit cost of ***c***. In our setting, the source of production risk is the weather conditions (**w)** whose distribution is given by w∼χ(w|ω), where ω is the micro-climate variables such as drought shock. This distribution is exogenous to the farmer's action. This is the only source of risk we considered; prices ***p*** and cost of production c&r are assumed to be non-random as farmers are price-takers in both input and output markets.

To capture the riskiness of the production process, we followed the approach of Di Falco and Chavas, 2006, Antle, 1983, and Zhang and Antle (2016). In particular, we captured the risk component of the production function by introducing the variance and skewness of maize yield through the moment-based approach as follows:

(1) $$E[{g\left(x,s,e,\;w\right)-f_{1}\left(x,s,e,\;w\right))}^{k}]=f_{k}\left(\text{x},\text{s},\text{e},\text{w},\beta_{k}\right)\forall k\geq 2$$

where $f_{1}\left(\cdot \right)=E(g(x,s,e,w)$ represents the mean of the production function. Given the above equation, the first moment (mean) of the production function is defined as:

(2) $$E\left[g\left(x,s,e,w\right)\right]=f_{1}\left(x,s,e,w,\beta_{1}\right)-cs-rx=\mu_{1}$$

Similarly, the second moment (variance) of the production function is defined as:

(3) $$E\left[(g\left(x,s,e,w\right)-E{\left(g\left(x,s,e,w\right))\right)}^{2}\right]=\mu_{2}$$

and the third moment (skewness) of the production function is defined as:

(4) $$E\left[(g\left(x,s,e,w\right)-E{\left(g\left(x,s,e,w\right))\right)}^{3}\right]=\mu_{3}$$

As shown by Antle (1987), the specification in Eqs. (2), (3), (4) can be further expressed as a function of all the moments of the production function using the third order Taylor approximation of the expected utility function as:

(5) $$E\left[u\left(\pi \right)\right]=f_{1}\left(x,s,e,w,\beta_{1}\right),f_{2}\left(x,s,e,w,\beta_{2}\right),\;f_{3}\left(x,s,e,w,\beta_{3}\right)=\left(\mu_{1},\mu_{2},\mu_{3}\right)\text{.}$$

where π is the net return from production. Since the farmers are risk-averse they maximize the expected utility of net returns from maize production in the following way:

(6) $$E\underset{xs}{max}E\left[u\left(\pi \right)\right]=u\left(\mu_{1},\mu_{2},\mu_{3}\right)\text{.}$$

The optimum condition for the adoption of DTMVs in elasticity form is then given by:

(7) $$\mu_{1}^{*}-\frac{cs}{\mu_{1}}-\frac{1}{2}\left(\frac{u^{\prime \prime }\left(\pi \right)}{u^{\prime }\left(\pi \right)}s_{2}\right)U_{2}^{*}+\frac{1}{6}\left(\frac{u^{\prime \prime \prime }\left(\pi \right)}{u^{\prime }\left(\pi \right)}s_{3}\right))U_{3}^{*}=0$$

where $\mu_{j}^{*}=\frac{\partial u_{j}}{\partial s}$, $s_{2}$ is the variance (or second central moment) of (π) and $s_{3}$ is the skewness (third central moment) of π (Antle, 1987, Di Falco and Chavas, 2006). From the above optimal condition, $\mu_{1}^{*}-\frac{cs}{\mu_{1}}$ captures the marginal net return of choosing DTMVs $\left(s^{*}\right)$ and the term $\left\{-\frac{1}{2}\left(\frac{u^{\prime \prime }\left(\pi \right)}{u^{\prime }\left(\pi \right)}s_{2}\right)U_{2}^{*}+\frac{1}{6}\left(\frac{u^{\prime \prime \prime }\left(\pi \right)}{u^{\prime }\left(\pi \right)}s_{3}\right))U_{3}^{*}\right\}$ depicts the marginal risk premium of adopting DTMVs (Chavas, 2004, Di Falco and Chavas, 2006, Zhang and Antle, 2016). Since DTMVs are risk-reducing, we would expect farmers to choose them, based on the marginal net return $\left(\mu_{1}^{*}-\frac{cs}{\mu_{1}}\right)$ and the marginal risk premium $\left(-\frac{1}{2}\left(\frac{u^{\prime \prime }\left(\pi \right)}{u^{\prime }\left(\pi \right)}s_{2}\right)U_{2}^{*}+\frac{1}{6}\left(\frac{u^{\prime \prime \prime }\left(\pi \right)}{u^{\prime }\left(\pi \right)}s_{3}\right))U_{3}^{*}\right)$.

**Fig. 1:**
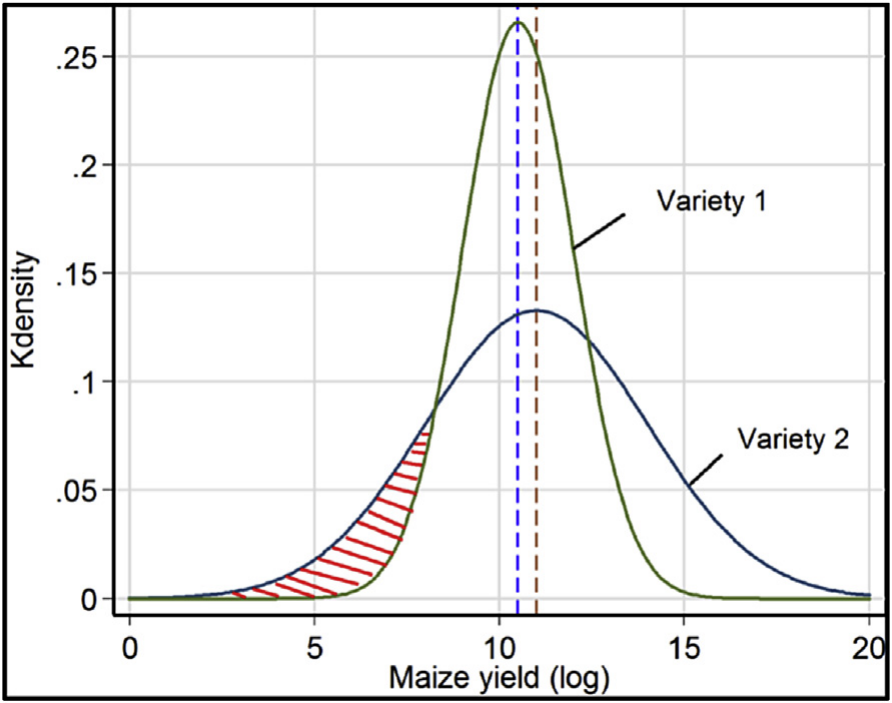
Adoption and variability of returns.

Fig. 1 presents the adoption decision of farmers based on the distribution of mean, variance, and skewness of maize yield. A profit-maximizing farmer adopts DTMVs when the returns from DTMVs are higher than the returns from non-DTMVs $\left(\pi_{dtmvs}-\pi_{ndtmvs}\right)\;\geq 0$. The term $\left(\pi_{dtmvs}-\pi_{ndtmvs}\right)$ captures the difference in mean returns with and without adoption. This is a necessary condition for adoption when the risk reduction effect of technologies is not considered. Since the mean return of variety 2 (red dotted vertical line) is higher than the mean return of variety 1 (blue dotted vertical line, Fig. 1), a profit-maximizing maize producer will adopt variety 2. However, since most farmers are risk-averse, adoption certainly depends on the second moment (variance) and the third moment (skewness) of yield. We assume that a typical maize producer, who strives to avoid food shortage due to crop failure, considers technologies that will reduce variability and downside risk in addition to making gains in productivity. Therefore, a risk-averse farmer is more likely to adopt variety 1 over variety 2, as the variance of variety 1 is smaller than that of variety 2. In addition, the probability of crop failure from variety 1 is smaller than the probability from variety 2 as shown in the shaded (red) region. Even with a lower mean value, the farmer may still adopt variety 1 so long as the gain from risk reduction (variability and crop failure) is larger than the loss from the mean output.

However, the trade-off between productivity gains and risk reductions is largely an empirical matter and depends on the risk preference of farmers, the type of technology under consideration, and the underlying insurance and credit markets. Nonetheless, the above condition suggests that adoption should reduce risk exposure (both variance and downside risk) as the technology is promoted as a variety with a superior performance (in terms of risk reduction) compared with currently available commercial varieties under both stress and optimal growing conditions (Fisher et al., 2015). The above theoretical model allows us to test the above assumptions explicitly by estimating the effect of adoption on the mean, variance, and skewness of maize yield.

## 3 Data sources and empirical strategy

### 3.1 Data sources

This study used household survey data collected by the International Institute of Tropical Agriculture (IITA) from November 2014 to February 2015 for the purpose of evaluating the impact of DTMVs on productivity, poverty, and food security in Nigeria. The study adopted a multistage stratified random sampling technique to collect data from a nationally representative sample of maize producers. The sampling strategy ensured that at least one maize farmer was sampled from each of the strata. The States in Nigeria were divided into homogenous sub-groups based on the number of hectares of land devoted to maize production. This gave five groups, from which 18 major maize-producing States were randomly selected. Following the National Bureau of Statistics (NBS) (2013) selection of the farming households for the Living Standards Measurement Study, we obtained the list of all Local Government Areas (LGAs) and Enumeration Areas (EAs) in each of the selected States from the National Population Commission. Following the NBS recommendation for a nationally representative data collection, we randomly selected 10% of the LGAs in each of the selected States and also satisfied the 95% confidence interval by selecting 5% of the total EAs per LGA.

Finally, five farming households were randomly selected from the households in each of the selected EAs. Before the actual process of data collection two pre-tests of the survey instrument were conducted This was designed to collect detailed information on a range of individual and socio-economic attributes, asset holdings and poverty measures, adoption of DTMVs, household expenditure on food and non-food items, output of maize and other notable crops, income from various sources, occurrence of drought shocks, membership in formal and informal associations, and access to credit and extension, among others. To minimize the errors usually encountered with the use of paper questionnaires, the data for this study were collected electronically using the “***surveybe****”* software.

### 3.2 Empirical strategy

We employed a simple empirical approach based on our theoretical framework to understand farmers' adoption decisions about DTMVs as well as the risk reduction roles of adoption. We first started our specification using a moment-based approach (Antle, 1983) in which the effect of adoption is directly incorporated in the first moment (mean), second moment (variance), and third moment (skewness which shows downside risk) of the maize production function in the following manner.

(8) y=f(s,x,v,e,w,ϕ)+u

where y is the logarithm of maize yield and s refers to the adoption of DTMVs, which takes on a value of 1 if a farmer adopts DTMVs and zero otherwise. Furthermore, ***x*** captures inputs of production other than DTMVs; v consists of a vector of socio-economic farm characteristics and social capital variables; ***e*** includes a set of village dummies, and w is weather variables. Finally, φ is a vector of parameters to be estimated and ***u*** is the error term. To capture the variability (variance) of maize yield, the estimated errors from the above equation were squared and regressed on the same explanatory variables to estimate the second moment of net return.

(9) $$u^{2}=f\left(s,x,v,e,w,\vartheta \right)+\hat{u}$$

Finally, by using the estimated errors raised to the power of three, the effect of adoption on downside risk (crop failure) was estimated as follows.

(10) $$u^{3}=f\left(s,x,v,e,w,\theta \right)+\tilde{u}$$

However, examining the causal effect of adoption on potential outcome indicators was not a trivial matter due to endogeneity bias as a result of observed and unobserved heterogeneities between adopters and non-adopters. Parameter estimates of Eqs. (8), (9), (10) could therefore be biased. We employed an endogenous switching regression (ESR) approach that accounts for both observed and unobserved sources of bias (Lokshin and Sajaia, 2004). We assumed that a particular farmer would consider adopting DTMVs if the expected benefit from adoption (in terms of productivity and welfare gain as well as the reduction of exposure to risk) is positive (Huang et al., 2015). If we allow the gain from adoption to be $A_{i}^{\text{*}}$, then $A_{i}^{\text{*}}$>0 implies that the benefit from adoption is greater than that of non-adoption. The latent variable $\left(A_{i}^{\text{*}}\right)$ can therefore be expressed in the following manner:

(11) $$A_{i}^{\text{*}}=f\left(s,x,v,e,w,z,\gamma \right)+\mu_{i}\;\mathrm{with}\;A=1\{A_{i}^{\text{*}}>0$$

where the vector z represents our instruments (variables that affect the decision to adopt DTMVs but not the outcome indicators) and γ is a vector of parameters to be estimated. The outcome function conditional on adoption can then be specified as an ESR model in the following manner.

(12) $$Regime1:Y_{1i}=f\left(\;s,x,v,e,w,\beta_{1}\right)+\;\varepsilon_{1i}\;\text{if}\;A_{i}=1$$

(13) $$Regime2:Y_{2h}=f\left(s,x,v,e,w,\beta_{2}\right)+\;\varepsilon_{2i}\;\text{if}\;A_{i}=0$$

where $Y_{1i}$ represents the yield for adopters and $Y_{2i}$ for non-adopters and $\varepsilon_{i}$ is the error term of the outcome variable. Identification of the ESR model requires at least one additional variable as an instrument in Eq. (11). In our setting, adoption of DTMVs can be endogenous as adopters may share common unobserved characteristics, such as entrepreneurship skills and management ability, which directly affect adoption and the productivity level of households (omitted variable bias). Following the literature (e.g., Bellemare, 2012), we used willingness to take risk for new maize varieties as an instrumental variable for our identification strategy. In the survey, we collected data on each respondent's willingness to take risks on seeds of new maize varieties during the planting stage. Since agricultural production is inherently risky, due to the lag between production decision and output realization, willingness to take risk on new maize varieties captures unobserved variations among respondents' respective marginal utilities derived from the use of new varieties (Bellemare, 2012). As such, it controls for variations in the different sources of unobserved heterogeneity among respondents (e.g., time preference, risk preferences, entrepreneurship skill of the farmer, etc.) that affect the decision to adopt DTMVs. The variable willingness to take risk was measured by a dummy variable which takes on a value of 1 if the respondent is willing to try any type of new maize varieties and zero, otherwise.

The error terms in the selection Eq. (11) and the outcome Eqs. (12), (13) are assumed to have a trivariate normal distribution with mean zero and covariance matrix (Ω) in the following manner:

$$Ω=\;\left[\begin{array}{ccc} \sigma_{\mu }^{2} & \sigma_{1\mu } & \sigma_{2\mu }\\ \sigma_{\text{μ}1} & \sigma_{1}^{2} & .\\ \sigma_{\text{μ}2} & . & \sigma_{2}^{2} \end{array}\right]$$

where $\sigma_{\mu }^{2}=var\left(\mu_{i}\right)$, $\sigma_{1}^{2}=var\left(\varepsilon_{1}\right)$, $\sigma_{2}^{2}=var\left(\varepsilon_{2}\right)$, $\sigma_{1\mu }=cov\left(\mu_{i},\;\varepsilon_{1}\right)$, $\sigma_{2\mu }=cov\left(\mu_{i},\varepsilon_{2}\right)$ Further, $\sigma_{\mu }^{2}$ is estimable up to a scale factor and can be assumed to be equal to 1 (Maddalla, 1983) and $cov\left(\varepsilon_{1},\varepsilon_{2}\right)$ is not defined as $Y_{1}$ and $Y_{2}$ cannot be observed simultaneously. Moreover, the correlation between the error term of the selection equation and the outcome equation is not zero (i.e., $corr\left(\mu_{i},\;\varepsilon_{1}\right)\neq 0\&corr\left(\mu_{i},\varepsilon_{2}\right)\neq 0$) which creates selection bias. ESR addresses this selection bias by estimating the inverse mills ratios (${\text{λ}}_{1i}$ and ${\text{λ}}_{2i}$) and the covariance terms ($\sigma_{1\mu }$ and $\sigma_{2\mu }$) and including them as auxiliary regressors in Eqs. (12), (13). If $\sigma_{1\mu }$ and $\sigma_{2\mu }$ are significant, we reject the absence of selection bias. The ESR model estimates can then be used to estimate ATT (Average treatment effect on treated households) and ATU (Average treatment effect on untreated households) as follows:

(14) $$E\left(Y_{1i}\text{|}A_{i}=1\right)=f\left(s,x,v,e,w,\beta_{1}\right)+{\text{λ}}_{1i}\sigma_{1\mu }$$

(15) $$E\left(Y_{2i}\text{|}A_{i}=0\right)=f\left(s,x,v,e,w,\beta_{2}\right)+{\text{λ}}_{2i}\sigma_{2\mu }$$

(16) $$E\left(Y_{2i}\text{|}A_{i}=1\right)=f\left(s,x,v,e,w,\beta_{2}\right)+{\text{λ}}_{1i}\sigma_{2\mu }$$

(17) $$E\left(Y_{1i}\text{|}A_{i}=0\right)=f\left(s,x,v,e,w,\beta_{1}\right)+{\text{λ}}_{2i}\sigma_{1\mu }$$

The ATT and ATU are then defined as:

(18) $$\text{ATT}=E\left(Y_{1i}|A_{i}=1\right)-E\left(Y_{2i}|A_{i}=1\right)$$

(19) $$\text{ATU}=E\left(Y_{1i}|A_{i}=0\right)-E\left(Y_{2i}|A_{i}=0\right)$$

The above estimates of ATT provide the effect of adoption on mean maize yield. However, given our objective of examining the role of adoption on risk exposure, we extended the above specification to capture the role of adoption on the variance and skewness of maize yield (to capture downside risk, such as crop failure. Following the same logic of Eqs. (18), (19), estimates of ATT and ATU for the second moment of maize yield (variance) were calculated as:

(20) $$\mathrm{ATT}=E\left(u_{1i}^{2}\text{|}A_{i}=1\right)-\text{E}\left(u_{2i}^{2}\text{|}A_{i}=1\right)$$

(21) $$\mathrm{ATU}=E\left(u_{1i}^{2}\text{|}A_{i}=0\right)-\text{E}\left(u_{2i}^{2}\text{|}A_{i}=0\right)$$

Similarly, estimates of ATT and ATU for the third moment of maize yield (skewness) will be calculated as:

(22) $$\text{ATT}=E\left(u_{1i}^{3}\text{|}A_{i}=1\right)-\text{E}\left(u_{2i}^{3}\text{|}A_{i}=1\right)$$

(23) $$\text{ATU}=E\left(u_{1i}^{3}\text{|}A_{i}=0\right)-\text{E}\left(u_{2i}^{3}\text{|}A_{i}=0\right)$$

In the context of ESR the error terms are assumed to be normally distributed. Hence, by construction the distribution is symmetric and the skewness is zero. Therefore, we ran an OLS specification and obtained the error terms and used them as dependent variables in the ESR specification for estimating the distribution of variance and skewness.

### 3.3 Descriptive statistics

#### 3.3.1 Outcome indicators

Table 1 presents the descriptive statistics of all outcome indicators and controls used for our analysis. The outcome indicators include per-capita expenditure (food, non-food, and total), maize yield, poverty headcount ratio, and seasonal food scarcity. For poverty, we followed the approach of Foster et al. (1984), and used per-capita total expenditure to determine households' poverty status. Formally, the headcount ratio ($P_{0}$) is calculated as:

(24) $$P_{0}=\frac{1}{N}\sum_{i=1}^{N}I\left(X_{p}<z\right)$$

where $X_{p}$ is the per capita total expenditure and N is the relevant population size. z is a poverty line. I(·) is an indicator function which takes on a value of 1 for $X_{p}<z$ and a value of zero for $X_{p}\geq z$. Our final outcome indicator, seasonal food scarcity, was measured by a dummy variable which takes on a value of 1 if the household did not have enough food to eat in any of the preceding 12 months and zero otherwise.

**Table 1:** Descriptive statistics by adoption status for DTMVs.

	Full sample (N = 2084)	Adopters (N = 592)	Non-adopters (N = 1492)	Mean difference
Maize yield (kg/ha)	2127	2502	1978	524***
Per capita food expenditure (₦)	51211	59481	47968	11513**
Per capita non-food expenditure (₦)	55429	67594	50657	16397***
Per capita total expenditure (₦)	106641	127076	98625	28450***
Poverty headcount ratio (1 = poor)	0.72	0.69	0.73	−0.04*
Food scarcity (1 = yes)	0.176	0.13	0.19	−0.06***
Household size	7.29	7.8	7.1	0.7***
Education (years of schooling)	7.3	6.9	7.5	−0.6**
Age	48.7	49.4	48.3	1.1*
Sex (1 = male, 0 = otherwise)	0.91	0.907	0.91	−0.006
Distance from seed market (km)	17.35	16.36	17.75	−1.4***
Willingness to take risk (1 = yes)	0.27	0.36	0.24	0.12*
Total value of assets (₦)	532411	556995	522657	34337***
Roofing material of the house (1 = has a sheet)	0.88	0.93	0.86	0.08***
Land tenure (1 = has tenure, 0 = otherwise)	0.87	0.88	0.86	0.02
Plot size (ha)	2	2.48	1.83	0.65
Drought shock (1 = experienced drought, 0 = otherwise)	0.30	0.81	0.14	0.67***
Member in informal associations (1 = yes, 0 = no)	0.68	0.62	0.70	−0.08***
Number of years of residence in the village	42.11	42.85	41.82	0–1.03
Access to electricity (1 = yes, 0 = otherwise)	0.45	0.49	0.44	0.05*
Labour (man-days)	102.4	116.1	97	19.1*
NPK fertilizer (kg)	206.3	303.8	167.5	136.2***
Urea fertilizer (kg)	106.5	166.3	83	83.3***
Use of pesticide (1 = yes)	0.44	0.38	0.46	−0.8***
Use of herbicide (1 = yes)	0.81	0.78	0.82	−0.04**
Good soil (1 = yes)	0.76	0.82	0.74	0.08***
Medium soil (1 = yes)	0.21	0.16	0.22	−0.06***
Poor soil (1 = yes)	0.03	0.02	0.04	−0.02
Use of soil and water conservation (1 = yes)	0.45	0.42	0.47	−0.05**
Men managed plots (1 = yes)	0.67	0.66	0.67	0.013
Women managed plots (1 = yes)	0.07	0.06	0.07	−0.01
Jointly managed plots (1 = yes)	0.26	0.28	0.26	0.02
Row planting (1 = yes)	0.65	0.60	0.66	−0.06***
Intercropping (1 = yes)	0.35	0.38	0.34	0.04

*** p < 0.01, ** p < 0.05, * p < 0.1.

**Fig. 2:**
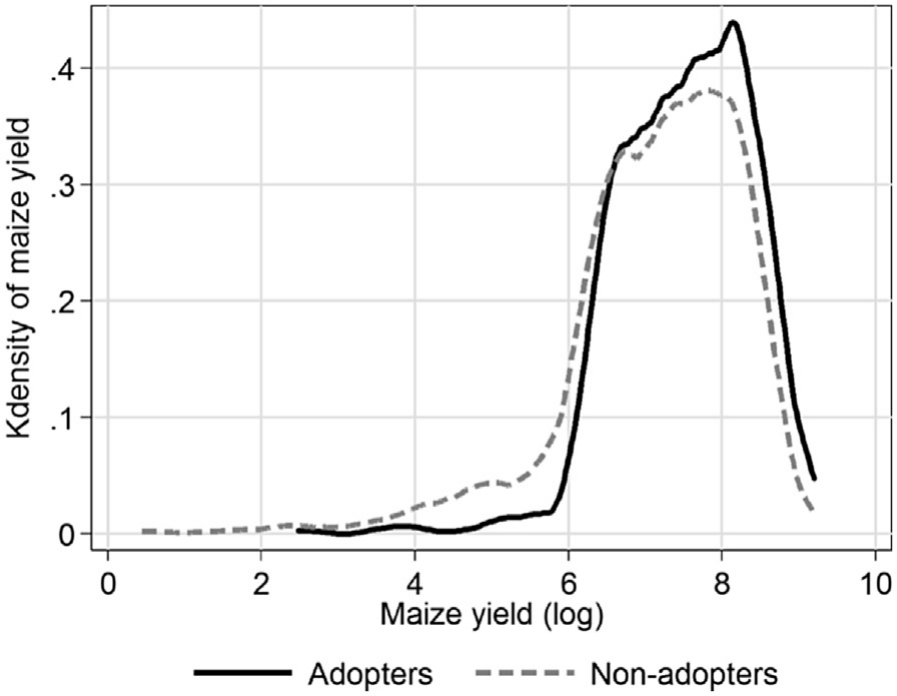
Distribution of maize yield among adopters and non-adopters.

For all outcome indicators, we assessed the existence of any significant differences in the means between adopters and non-adopters using *t*-test and found statistically significant differences for all outcome indicators. However, it is worth noting that these results do not show causality. Fig. 2 reports the distribution of maize yield between adopters and non-adopters and shows that the average yield was slightly higher among adopters. In addition, the left tail of the distribution further suggests that the skewness of maize yield was lower among adopters. Further the Kolmogorov–Smirnov equality-of-distributions test suggested that the two distributions are different.^2^

#### 3.3.2 Socio-economic and plot variables

In addition to outcome variables of interest, Table 1 also presents the description of the main socio-economic and plot-level variables. Our main variable of interest, adoption of DTMVs, was measured by a dummy variable that takes on a value of 1 if a given farmer adopts them and zero otherwise. Based on economic theory, we included control variables at household and village levels. These include household characteristics such as age, household size, and education, membership in different social groups, occurrence of drought shocks as well as wealth indicators such as land size, the value of farms, and non-farm assets. We assumed that the above key household characteristics affected farmers' adoption decisions as well as their productivity and ultimate poverty status. In addition to household characteristics, we also included location dummies to account for common market and weather shocks as well as the general economic condition at the State level. Table 1 showed that the average household size is about 7.3 members for the whole sample. While comparing household size between adopters (7.8 members) and non-adopters (7.1 members), we found significant differences between the two groups. About 90% of the household heads were male and their average age was about 48 years. In terms of self-reported shocks, about 30% of the respondents had experienced drought shocks. However, about 81% of adopters have reported experiencing drought shocks while only 14% of non-adopters have reported experiencing drought shocks. This results suggests that farmers adopt DTMVs in response to drought shocks. Table 1 further presented the difference in means between adopters and non-adopters. The results revealed significant systematic differences between them in terms of several key socioeconomic characteristics. Clearly, a simple comparison of adopters and non-adopters in terms of the main outcomes of interest without accounting for the differences in observable characteristics would bias the estimated impacts of DTMVs.

Table 1 further shows input allocation decisions of adopters and non-adopters. The descriptive statistics revealed that the application of chemical fertilizer was significantly higher among adopters compared with non-adopters. Similarly, labour use (measured in man-days) was significantly higher among adopters than non-adopters. The use of pesticide and herbicide was also relatively high.

## 4 Empirical results

### 4.1 OLS results

Table 2 presents the effects of adoption on mean, variance, and downside risk exposure.^3^ Estimated results showed that adoption has a statistically significant effect on productivity, with adoption increasing maize yields by 11%. Moreover, adoption has a negative and statistically significant effect on the variance of maize yield. This result showed that adoption reduced the variability of maize yield. However, variance does not distinguish between unexpected good and bad outcomes. In the context of food insecure smallholders, avoiding unexpected bad outcomes is very important. As a result, we reported the effect of adoption on downside risk (skewness of maize yield). The regression results for the skewness function show that adoption significantly reduced exposure to downside risk as the coefficient on adoption is positive and statistically significant. As such, adoption can serve as insurance for farmers by reducing the risk of crop failure.

**Table 2:** OLS estimates of the effects of adoption on mean, variance and skewness of maize yields.

	Average yield	Variance of yield	Skewness of yield
Adoption	0.111**	−0.354***	0.993***
	(0.0491)	(0.0936)	(0.384)
Other controls	Yes	Yes	Yes
Location dummies	Yes	Yes	Yes
N	2084	2084	2084

Robust standard errors are reported in parentheses, *** p < 0.01, ** p < 0.05. **Other controls include**: education, sex, age, Distance from seed source, use of labour, fertilizer, pesticide, and herbicide, plot management dummies, soil fertility dummies, management practice dummies, drought shock. **Location dummies were** North-West, South-South, North-Central, North-East and South-West.

Our results suggested that DTMVs have a win-win outcome as these varieties increased yield and reduced variability and the probability of crop failure. However, these results should be interpreted with caution as we did not control for the potential endogeneity of adoption. In the next section, we present an ESR model where we controlled for unobserved heterogeneity.

### 4.2 Endogenous switching regression results

Herein, we present our counterfactual analysis. Unlike the OLS results presented in the previous section, the ESR model accounted for both observable and unobservable sources of heterogeneity between adopters and non-adopters.^4^ We used the ESR model results to compare the distribution of mean, variance, and skewness of maize yield with and without adoption. In particular, we investigated the distribution of mean, variance, and skewness of maize yield among adopters (ATT) and current non-adopters of DTMVs (ATU). The results revealed that adoption increases average yield by 13.3%. This result shows that maize yield among adopters would have declined by 13.3% if they had not adopted DTMVs. Similarly, the maize yield of non-adopters would have increased by 9.4% if they had adopted DTMVs. ATT results suggest that adoption significantly increased productivity and hence food security among current adopters. In addition, ATU results suggested that further dissemination efforts will increase resilience and food security in the region as a significant share of farmers are still non-adopters (for this group, productivity would have increased by 9.4% had they adopted DTMVs).

Our results were in line with the regional on-farm trial values in yield gain as IITA reported a significantly higher yield for DTMVs compared with commercial hybrid maize varieties and local varieties in Nigeria. In particular, their on-farm regional trial data showed that DTMVs out-yielded commercial hybrid maize varieties by a minimum of 6% and local varieties by 33%. Table 3 also presents ATT and ATU on the variance and skewness of maize yield. Adoption was found to significantly reduce exposure to risk. For instance, the risk (variance) faced by adopters would have increased by 0.79 units (about 53%) if they had not adopted DTMVs. Similarly, the downside risk (crop failure) faced by adopters would have increased by 2.2 units (about 81%) if they had not adopted DTMVs. These results underscored the fact that DTMVs are both yield enhancing and risk reducing. The ATU results further suggested that non-adopters would have benefited significantly in terms of reduction to risk exposure if they had adopted DTMVs. In particular, the variance they face would have declined by 23% and downside risk by 30%. These results further affirm that further dissemination of DTMVs to non-adopters will be crucial to increase food production, resilience, and food security.

**Table 3:** Effect of adoption on the mean, variance and skewness of maize yield.

Outcome variables	Farm household type and treatment effect	Decision stage		Effect of adoption	Change (%)
		To adopt	Not to adopt		
Average maize yield	Adopters (ATT)	7.52	6.64	0.88***	13
	Non-adopters (ATU)	7.82	7.14	0.67***	9.4
Average variance (risk)	ATT	0.69	1.49	−0.79***	−53
	ATU	0.91	1.18	−0.27***	−22.9
Average skewness (downside risk exposure)	ATT	−0.51	−2.7	2.2***	81
	ATU	−1.26	−1.8	0.54***	30

*** p < 0.01, ** p < 0.05, * p < 0.1.

**Fig. 3:**
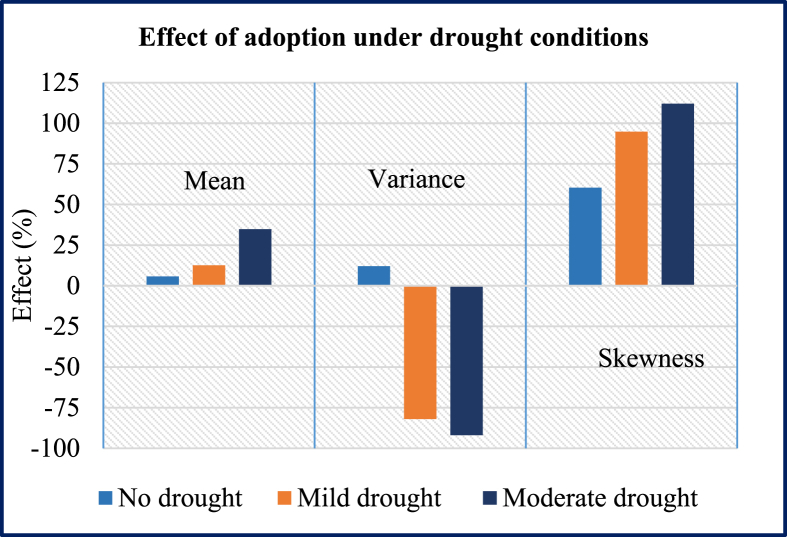
Effect of adoption under drought conditions.

To add further novelty, we estimated heterogeneity effects by classifying villages into three main groups based on drought severity index. The drought severity index was calculated using the African flood and drought monitor data from Princeton University. Based on the drought severity index, we found that about 38% of the villages have not experienced any drought while 35% and 27% of the villages experienced mild and moderate drought conditions, respectively.^5^ Fig. 3 below reports estimated effects for each respective group. We found a small but positive effect on mean yield (about 5.7%) and an insignificant effect on the variance and skewness of maize yield under normal (no drought) condition. However, under mild and moderate drought conditions, we found significant effects on the mean, variance and skewness of maize yield, suggesting that DTMVs were important adaptation strategies to drought stress.^6^

Next, we present the welfare effects of adoption. We used per-capita food, non-food, and total expenditure, expressed in natural logarithms, poverty headcount ratio, as well as food security indicators to examine the role of adoption on welfare. The results are based on our counterfactual analysis using ESR model results for welfare outcome indicators.^7^ The results (Table 4) show that adoption had a significant effect on all welfare indicators. Our result showed that if adopters of DTMVs had not adopted them, the observed poverty rate in the sample would have been higher by 12.9%. This result suggested that the gain in maize productivity due to adoption had eventually reduced the incidence of poverty by 12.9%. Similarly, estimates of ATU suggested that the incidence of poverty would have declined by 26.2% for current non-adopters. In terms of consumption expenditures, the result shows that per capita food expenditure of adopters would have declined by 5.9% and total consumption expenditure by 7.2% had they not adopted (i.e., they would have had 5.9% less expenditure per capita on food and 7.2% less on total consumption). ATU results further suggest that per capita food expenditure of non-adopters would have increased by 9% and total consumption by 8%, had they adopted DTMVs.

**Table 4:** Effect of adoption on poverty and food security.

Outcome variables	Farm household type and treatment effect	Decision stage		Adoption effects	Change (%)
		To adopt	Not to adopt		
Per-capita total expenditure	Adopters (ATT)	11.51	10.74	0.77***	7.2
	Non-adopters (ATU)	12.17	11.27	0.90***	7.98
Per-capita food expenditure	ATT	10.74	10.14	0.60***	5.9
	ATU	11.39	10.44	0.945***	9
Per-capita non-food expenditure	ATT	10.75	9.83	0.92***	9.4
	ATU	11.65	10.26	1.4***	13.6
Food scarcity^a^	ATT	0.03	0.185	−0.155***	83.8
	ATU	0.15	0.64	−0.49***	76.5
Poverty headcount ratio	ATT	0.704	0.808	−0.104***	12.9
	ATU	0.535	0.725	−0.19***	26.2

*** p < 0.01, ** p < 0.05, * p < 0.1.

a For food security, we used switch probit command of stata and hence values are probabilities.

When it comes to food scarcity, ATT and ATU results show the importance of adopting DTMVs. In particular, if adopters had not adopted, the probability of experiencing seasonal food scarcity would have increased by 83.8%. Similarly, if non-adopters had adopted, the probability of experiencing seasonal food scarcity would have declined by 76.5%. Given the significant positive gains in welfare among current adopters and expected benefits for non-adopters, should they adopt DTMVs further efforts to increase dissemination will lead to higher impacts in terms of improving total food production and food security in Nigeria.^8^

After establishing the causal effect of adoption on a range of outcomes of interest, we then calculated the number of individuals who have managed to overcome poverty as a result of adoption, following the procedure of Zeng et al. (2015). In particular, we combined information on parameter estimates on poverty headcount ratio as well as adoption rates to determine the number of individuals lifted above the poverty line as a result of adopting DTMVs. According to FAOSTAT (2014), about 5.85 million ha were allocated for maize production in Nigeria in 2014. Taking into account the average maize land among adopters, the total number of maize producing farmers in Nigeria is estimated to be around 9.2 million. Given an adoption rate of 28%, a parameter estimate of 12.9% on poverty headcount ratio, and the above-mentioned number of maize farmers, close to 0.27 million farmers have managed to escape poverty as a result of adopting DTMVs.^9^ Given the average family size of 7.8 among current adopters in Nigeria (See Table 1), the total number lifted above the poverty line becomes 2.1 million individuals^10^.

## 5 Conclusions and implications

Using household and plot-level data from rural Nigeria, this paper assessed the impact of adopting DTMVs on the productivity, risk exposure, and welfare among maize farming households. With the aim of providing consistent estimates of the impact of adoption on productivity and welfare outcomes, the study employed an endogenous switching regression approach that controls for both observed and unobserved heterogeneity between adopters and non-adopters. In doing so, the study not only evaluated the extent to which adoption affected productivity and welfare outcomes but also examined its implication on risk exposure focusing on variance and downside risk. Our main results are summarized as follows: first, adoption increased yield. In particular, if adopters had not adopted DTMVs, their yield would have declined by 13.3%. Secondly, without adoption the level of variance among adopters could have increased by 53% and downside risk exposure by 81%. Furthermore, ATU results suggested that for non-adopters, the variance they face would have declined by 23% and downside risks by 30% if they had adopted DTMVs. Thirdly, the productivity-enhancing and risk-reducing roles of DTMVs have a significant effect on household welfare. In particular, our results showed that per capita food expenditure of adopters would have been lower by 5.9% and total consumption expenditure by 7.2%, had they not adopted DTMVs. Moreover if adopters had not adopted, poverty would have been 12.9% higher and the probability of seasonal food scarcity would have increased by 84%. Altogether, adoption had a “win-win” outcome by improving productivity and reducing risk. These results re-affirmed that interventions against drought stress through crop genetic improvements will have a paramount role to play in terms of enhancing food security and reducing the farmer's exposure to drought risk. The results further underscored that future dissemination efforts will be crucial as current adoption rates are quite low, despite the reported benefits.

## Footnotes

1 We included skewness of yield since the variance does not distinguish between unexpected bad events and unexpected good ones. By capturing the skewness of maize yield, we can examine the effect of adoption of DTMVs on downside risk (e.g., a decrease in the probability of crop failure).

2 Equity of the two distributions is rejected at 1% as the p-value on Combined K-S is less than 0.01.

3 The full estimated results are available in Table 1 of the online supplementary material.

4 The full ESR results are presented in Table 2 in the online supplementary material.

5 The drought severity index conditions are normally categorized into five groups (incipient drought, mild drought, moderate drought, severe drought and extreme drought). However, in our data, we were able to identify only no drought, mild drought and moderate drought conditions.

6 Extended discussion on this results is available in the online supplementary material.

7 Note that ESR results for total expenditure, food expenditure, and non-food expenditure are not reported here owing to space limitations but are available upon request.

8 Robustness checks using matching techniques are presented in the online supplementary material.

9 Calculated as 9.2*0.28*0.104.

10 Calculated as (0.27*7.8). We assumed perfect income distribution within a family.

## References

[bib1] Antle J. (1983). Testing the stochastic structure of production: a flexible-moment based approach. J. Bus. Econ. Stat..

[bib2] Antle J.M. (1987). Econometric estimation of producers' risk attitudes. Am. J. Agric. Econ..

[bib3] Bellemare Marc F. (2012). As You Sow, so Shall You Reap: the Welfare Impacts of Contract Farming. World Dev..

[bib4] Bryan E., Ringler C., Okoba B., Roncoli C., Silvestri S., Herrero M. (2013). Adapting agriculture to climate change in Kenya: household strategies and determinants. J. Environ. Manag..

[bib5] Chavas J.P. (2004). Risk Analysis in Theory and Practice.

[bib6] Collier P., Conway G., Venables T. (2008). Climate change in Africa. Oxf. Rev. Econ. Policy.

[bib7] Di Falco S., Chavas J.P. (2006). Crop genetic diversity, risk exposure, and food security in the highlands of Ethiopia. Am. J. Agric. Econ..

[bib8] Fisher M., Abate T., Lunduka R.W., Asnake W., Alemayehu Y., Madulu R.B. (2015). Drought tolerant maize for farmer adaptation to drought in sub-Saharan Africa: determinants of adoption in eastern and southern Africa. Clim. Change.

[bib23] Food and Agriculture Organization of the United Nations (2014). FAOSTAT Statistics Database. http://www.fao.org/faostat/en/#data/QC.

[bib9] Foster J., Greer J., Thorbecke E. (1984). A class of decomposable poverty measures. Econometrica.

[bib10] Haile M.G., Wossen T., Tesfaye K., von Braun J. (2017). Impact of climate change, weather extremes, and price risk on global food supply. Econ. Disasters Clim. Change.

[bib11] Huang J., Wang Y., Wang J. (2015). Farmers' adaptation to extreme weather events through farm management and its impacts on the mean and risk of rice yield in China. Am. J. Agric. Econ..

[bib12] Hyman G.G., Fujisaka S., Jones P.G., Wood S., de Vicente C., Dixon J. (2008). Strategic approaches to targeting technology generation: assessing the coincidence of poverty and drought-prone crop production. Agric. Syst..

[bib13] Kostandini G., La Rovere R., Abdoulaye T. (2013). Potential impacts of increasing average yields and reducing maize yield variability in Africa. Food Policy.

[bib14] Koundouri P., Nauges C., Vangelis T. (2006). Technology adoption under production uncertainty: theory and application to irrigation technology. Am. J. Agric. Econ..

[bib15] La Rovere R., Abdoulaye T., Kostandini G., Guo Z., Mwangi W., MacRobert J., Dixon J. (2014). Economic, production and poverty impacts of investing in maize tolerant to drought in Africa. J. Dev. Areas.

[bib16] Lokshin M., Sajaia Z. (2004). Maximum likelihood estimation of endogenous switching regression models. Stata J..

[bib17] Maddalla G.S. (1983). Limited Dependent and Qualitative Variables in Econometrics.

[bib18] Schlenker W., Lobell D.B. (2010). Robust negative impacts of climate change on African agriculture. Environ. Res. Lett..

[bib19] Tambo J., Abdoulaye T. (2012). Climate change and agricultural technology adoption: the case of drought tolerant maize in rural Nigeria. Mitig. Adapt. Strateg. Glob. Change.

[bib20] Wossen T., Di Falco S., Berger T., McClain W. (2016). You are not alone: social capital and risk exposure in rural Ethiopia. Food Secur..

[bib21] Zeng D., Alwang J., Norton G., Shiferaw B., Jaleta M., Yirga C. (2015). Ex post impacts of improved maize varieties on poverty in rural Ethiopia. Agric. Econ..

[bib22] Zhang H., Antle J. (2016). Assessing climate vulnerability of agricultural systems using high-order moments: a case study in the U.S. Pacific northwest. Selected Paper Prepared for Presentation at the 2016 Agricultural & Applied Economics Association Annual Meeting, Boston, Massachusetts, 31 July -2 August.

