# Supplementary material for: Measuring the impacts of adaptation strategies to drought stress: The case of drought tolerant maize varieties
Source: J Environ Manage. 2017 Dec 1;203:106–13. doi: 10.1016/j.jenvman.2017.06.058 (PMC5607453; doi:10.1016/j.jenvman.2017.06.058)
Supplement: Supplementary file 1 [file mmc1.docx]

# Online supplementary material

## OLS results

Table 1: OLS estimates on the effects of adoption on mean, variance and skewness of maize yields.

|  | Maize yield | Variance | Skewness |
| --- | --- | --- | --- |
| Adoption | 0.111** | -0.354*** | 0.993*** |
|  | (0.0491) | (0.0936) | (0.384) |
| Education | 0.00270 | -0.00491 | 0.00467 |
|  | (0.00434) | (0.00946) | (0.0433) |
| Age | 0.00913 | -0.0191 | -0.0690 |
|  | (0.0110) | (0.0212) | (0.0952) |
| Sex | 0.111 | -0.432 | 1.401 |
|  | (0.0962) | (0.271) | (1.431) |
| Distance from seed source | -0.000222 | 0.00299 | -0.0204 |
|  | (0.00233) | (0.00526) | (0.0218) |
| labour (log) | 0.179*** | -0.108 | 0.107 |
|  | (0.0317) | (0.0834) | (0.392) |
| Chemical fertilizer (log) | 0.0453*** | -0.102*** | 0.363** |
|  | (0.0117) | (0.0325) | (0.179) |
| Use of pesticide | 0.0658 | -0.0817 | 0.292 |
|  | (0.0503) | (0.130) | (0.660) |
| Use of herbicide | -0.0105 | 0.333*** | -1.318** |
|  | (0.0602) | (0.115) | (0.534) |
| Good soil | 0.166 | 0.178 | -0.674 |
|  | (0.127) | (0.189) | (0.631) |
| Medium soil | 0.0710 | 0.461** | -1.647** |
|  | (0.134) | (0.220) | (0.817) |
| Use of soil & water conservation | -0.186*** | 0.456*** | -1.605** |
|  | (0.0533) | (0.143) | (0.716) |
| Men managed plots | -0.00105 | 0.193 | -0.934 |
|  | (0.0982) | (0.227) | (1.079) |
| Jointly manged plots | 0.106 | 0.108 | -0.893 |
|  | (0.120) | (0.268) | (1.226) |
| Row planting | -0.0419 | 0.101 | -0.685 |
|  | (0.0619) | (0.132) | (0.585) |
| Intercropping | 0.116** | 0.579*** | -2.021** |
|  | (0.0563) | (0.160) | (0.827) |
| Irrigation | -0.101 | 0.307 | -1.251 |
|  | (0.0947) | (0.237) | (1.023) |
| Land tenure | -0.00313 | -0.106 | 0.747 |
|  | (0.0437) | (0.122) | (0.609) |
| Drought shock | -0.0596 | -0.0403 | 0.0622 |
|  | (0.0606) | (0.146) | (0.666) |
| North-West | 0.00170 | 0.167 | -1.125 |
|  | (0.111) | (0.226) | (0.933) |
| South-South | -0.621*** | 0.371 | -0.0600 |
|  | (0.172) | (0.331) | (1.351) |
| North-Central | -0.424*** | 0.0977 | -0.140 |
|  | (0.120) | (0.246) | (1.036) |
| North east | -0.238 | -0.0213 | -0.921 |
|  | (0.160) | (0.440) | (2.100) |
| South-west | 0.0627 | -0.338* | 0.548 |
|  | (0.112) | (0.196) | (0.772) |
| Observations | 2084 | 2084 | 2084 |

Robust standard errors are reported in parentheses, *** p<0.01, ** p<0.05, * p<0.1.

## Endogenous Switching regression results

We now turn to the results of our ESR analysis where we accounted for unobserved heterogeneity (selection and endogeneity bias) using ESR approach. ESR results for the selection equation are presented and discussed first to show the relevance of our instrument and to examine other determinants of adoption. We then present our ESR estimates on the determinants of maize yield for adopters and non-adopters. The selection equation where we examined the determinants of adoption is presented in the first column of Table 2. Among the socio-economic characteristics membership in social networks and years of residence have a significant effect on the probability of adopting DTMVs. Most importantly, experiencing a drought shock has a positive and significant effect on the probability of adopting DTMVs. This result suggests that farmers who are exposed to random drought shock are more likely to adopt DTMVs as an ex-ante risk reduction strategy. Other important determinants of adoption include the use of other complimentary technologies such as irrigation, soil and water conservation practices and other conventional inputs such as chemical fertilizer. The result reported in Table 2 further confirms that our instrument, willingness to try new maize varieties, has a positive and statistically significant effect on the probability of adopting DTMVs. This result suggests that our instrument is relevant. Estimated coefficients of the correlation terms $\rho_{1\mu}$ and $\rho_{0\mu}$*,* which measures the correlation between the error terms of the selection equation and the outcome equation are reported in Table 2, bottom row. The coefficient of $\rho_{0\mu}$ is negative and statistically significant while the coefficient of $\rho_{1\mu}$ is insignificant. This result underscores the presence of selection bias. As such, the results of our OLS estimates are biased and hence unobserved heterogeneity between adopters and non-adopters has to be taken into account. In addition, the negative and significant effect of $\rho_{0\mu}$ suggests that current non-adopters have higher average maize yield than a random individual from the sample while current adopters do no better or worse than a random individual^[[1]](#footnote-1)^.

Table 2: Determinants of adoption and maize yield

|  | Selection equation | | | Adopters | | Non-adopters | |
| --- | --- | --- | --- | --- | --- | --- | --- |
|  | Coefficient | | z-value | Coefficient | z-value | Coefficient | z-value |
| Education | -0.0014 | 0.0068 | | -0.0099 | 0.0094 | 0.0086 | 0.0055 |
| Age | -0.0198 | 0.0142 | | 0.0101 | 0.0187 | 0.0190 | 0.0142 |
| Age^2^ | 0.0002* | 0.0001 | | -0.0001 | 0.0002 | -0.0001 | 0.0001 |
| Distance from seed source | -0.0020 | 0.0039 | | -0.0009 | 0.0042 | 0.0002 | 0.0028 |
| Land tenure | 0.0882 | 0.0605 | | -0.0545 | 0.0636 | -0.0130 | 0.0584 |
| Household size | 0.0185 | 0.0082 | | -0.0022 | 0.0103 | 0.015** | 0.0071 |
| Value of farm assets | -0.0114 | 0.0157 | | 0.0158 | 0.0170 | 0.029*** | 0.0114 |
| Roofing material of the house | 0.1855 | 0.1247 | | -0.1107 | 0.1254 | 0.1233 | 0.0872 |
| Member to social networks | 0.17** | 0.0841 | | 0.0013 | 0.0783 | 0.0698 | 0.0672 |
| Village residence | -0.07*** | 0.0027 | | -0.0050 | 0.0032 | -0.005** | 0.0021 |
| Access to electricity | -0.0150 | 0.0728 | | -0.0193 | 0.0693 | 0.173*** | 0.0623 |
| Drought shock | 0.29*** | 0.0803 | | -0.0277 | 0.2526 | -0.23*** | 0.0792 |
| Men managed plots | -0.2109 | 0.1467 | | 0.0155 | 0.1328 | 0.1799 | 0.1272 |
| Jointly manged plots | -0.2093 | 0.1732 | | 0.0418 | 0.1890 | 0.296** | 0.1480 |
| Row planting | -0.2402 | 0.0871 | | 0.0111 | 0.1280 | 0.0327 | 0.0793 |
| Intercropping | 0.0634 | 0.0749 | | 0.154* | 0.0879 | 0.0397 | 0.0704 |
| Irrigation | -0.243* | 0.1266 | | 0.39*** | 0.1132 | -0.1981 | 0.1268 |
| Use of pesticide | -0.0850 | 0.0727 | | 0.21*** | 0.0776 | 0.0251 | 0.0639 |
| Use of herbicide | -0.1946 | 0.0975 | | 0.0983 | 0.1343 | 0.0138 | 0.0801 |
| Good soil | 0.1658 | 0.2429 | | 0.0520 | 0.2409 | 0.2073 | 0.1490 |
| Medium soil | 0.2662 | 0.2530 | | 0.0145 | 0.2093 | 0.0228 | 0.1594 |
| Use of SWC | -0.21*** | 0.0781 | | -0.0110 | 0.1020 | -0.18*** | 0.0690 |
| labour (log) | 0.40*** | 0.0614 | | 0.1397 | 0.1004 | 0.06* | 0.0335 |
| Chemical fertilizer (log) | 0.063*** | 0.0171 | | 0.082*** | 0.0301 | 0.036*** | 0.0135 |
| Willingness to try new maize variety | 0.36*** | 0.087 | |  |  |  |  |
| ${ln\sigma}_{1}$ |  |  | | 0.81 | 0.107 |  |  |
| $\rho_{1\mu}$ |  |  | | -0.38 | 0.46 |  |  |
| ${ln\sigma}_{0}$ |  |  | |  |  | 1.14*** | 0.028 |
| $\rho_{0\mu}$ |  |  | |  |  | -0.41*** | 0.06 |
| Wald chi2 | 98.2*** |  | |  |  |  |  |
| N | 2084 |  | |  |  |  |  |

*** p<0.01, ** p<0.05, * p<0.1.

These results suggest that farmers with above than average maize yields are less likely to adopt DTMVs. In addition, Table 2 presents the effect of conventional inputs and other socio-economic variables on the maize yield of adopters and non-adopters. The dependent variable, maize yield, is expressed in natural logarithm. We found that application of chemical fertilizer has a positive and statistically significant effect on maize yield of adopters and non-adopters. In particular, increasing the application of chemical fertilizer by 1% increases maize yields by 0.082% for adopters and 0.036% for non-adopters respectively^[[2]](#footnote-2)^. This result underscores that fertilizer response of DTMVs is much higher than traditional maize varieties. Similarly, increasing the application of labour by 1% increases maize yields among non-adopters by 0.06%. For other conventional inputs and socio-economic variables, the result shows that the direction and magnitude of parameter estimates on maize yield are different between adopters and non-adopters. Of particular interest is the effect of drought shock. We find that while drought shock did not significantly reduce maize yield of adopters, it did significantly reduce the maize yield of non-adopters. This implies that adoption of DTMVs serve as an ex-ante risk reducing strategy against the adverse effects of drought shock. A difference in yield response between adopters and non-adopters is also observed for pesticide use. Pesticide use has a statistically significant and positive effect on maize yield of adopters, but has small and insignificant effect for non-adopters.

## Robustness check using PSM and IPWRA

Table 7 reports the effect of adoption of DTMVs of productivity, risk exposure and welfare outcomes using propensity score matching (PSM) and inverse probability weighted regression approach (IPWRA) as a robustness check. The first column presents results using PSM while results in the second column show results of IPWRA specification. PSM results show that adoption increases productivity of maize by 14%.

Table 3: PSM and IPWRA results

|  | PSM | IPWRA |
| --- | --- | --- |
| Log of maize yield | 0.14* | 0.116** |
|  | (0.82) | (0.048) |
| Variance of maize yield | -0.79*** | -0.57*** |
|  | (0.137) | (0.082) |
| Skewness of maize yield | 1.02** | 1.22*** |
|  | (0.43) | (0.036) |
| Log of per-capita total expenditure | 0.67*** | 0.77** |
|  | (0.09) | (0.047) |
| Log of per-capita food expenditure | 0.49*** | 0.56*** |
|  | (0.103) | (0.048) |
| Log of per-capita non-expenditure | 0.84*** | 0.96*** |
|  | (0.091) | (0.065) |
| Subjective food insecurity | -0.0012 | -0.056 |
|  | (0.066) | (0.025) |

Robust standard errors are reported in parentheses, *** p<0.01, ** p<0.05, * p<0.1.

Estimated results are quantitatively similar while using IPWRA specification (adoption increases maize yield by 11.6%). The above two results underscores that adoption of DTMVs can significantly improve productivity. We also investigated the distribution of variance and skewness (downside risk) of maize yield using PSM and IPWRA approaches. PSM and IPWRA results on the variance and skewness of maize yields suggest that DTMVs reduce variance and the probability of crop failure. These results underscore that DTMVs are both yield enhancing and risk reducing varieties. Since the reliability of both PSM and IPWRA results depend on the quality of matching, we report the overall covariate balancing tests before and after matching in Table 8. The results reveal that the standardized mean difference for all covariates used in the PSM is reduced from 21.1% pre-matching to 5.3% post-matching. This result shows that matching reduces bias by about 74.9%. In addition, we rejected the joint significance of covariates post-matching (p-value=0.176) while the joint significance of covariates was not rejected before matching (p-value=0.0000). Moreover, due to matching, the pseudo-R2 declined from 19.2% to 2%.

Table 4: Propensity score matching quality test

|  | Pseudo R2 | LR X2 | p-value | Mean bias |
| --- | --- | --- | --- | --- |
| Before matching | 0.192 | 445.5 | 0.0000 | 21.1 |
| After matching | 0.02 | 25.7 | 0.176 | 5.3 |

The high total bias reduction, the insignificant p-values of the likelihood ratio test after matching, low pseudo-R2 and significant reduction in the mean standardized bias are indicative of successful balancing of the distribution of covariates between adopters and non-adopters. Consistent results while using OLS, PSM, IPWRA and IV regression approaches underlines the robustness of estimated effects of adopting DTMVS on productivity, risk exposure and welfare outcomes.

## Heterogeneity effects based on the incidence of drought

In order to provide, heterogeneity effects of adoption under different drought conditions, we use the drought severity index (DSI) (see, Mu *et al*. 2013). DSI values for all surveyed villages is presented in Table 5. As shown in Table 5, about 38% of the villages have not experienced any drought while 35% and 27% of the villages experienced mild and moderate drought conditions, respectively.

Table 5: classification of villages into different drought conditions based on DSI

| Type of drought | DSI | Share of villages (%) |
| --- | --- | --- |
| No drought | 0 | 38 |
| Incipient drought | -0.3 to -0.59 | 0 |
| Mild drought | -0.6 to -0.89 | 35 |
| Moderate drought | -0.9 to -1.19 | 27 |
| Severe drought | -1.2 to -1.49 | 0 |
| Extreme drought | <-1.5 | 0 |

Using the above DSI, values we grouped villages into three groups and estimated the effect of adoption on the mean, variance and skewness of maize production. Results are presented in Table 6. Our estimated effects for each respective group revealed a small positive effect on mean yield (about 5.7%) and an insignificant effect on the variance and skewness of maize yield under normal (no drought) condition. However, under mild and moderate drought conditions, we found significant effects on the mean, variance and skewness of maize yield, suggesting that DTMVs were important adaptation strategies to drought stress.

## Reference

Mu, Q., Zhao, M., Kimball, J. S., McDowell, N. G. and Running, S. W. (2013). A remotely sensed global terrestrial drought severity index. Bulletin of the American Meteorological Society, 94(1), 83-98.

1. ESR results for variance and skewness are not reported here due to space limitation. However, estimates are available from authors upon request [↑](#footnote-ref-1)
2. Coefficients are interpreted as elasticities as the dependent variable represents the logarithm of maize yield and fertilizer is transformed in logarithmic form. [↑](#footnote-ref-2)
